# Adaptation, Student Participation and Gradual Withdrawal by Researchers as Sustainability Strategies in the High School-Based Young and Active Intervention: School Coordinators’ Perspectives

**DOI:** 10.3390/ijerph181910557

**Published:** 2021-10-08

**Authors:** Stine Kjær Wehner, Tine Tjørnhøj-Thomsen, Katrine Sidenius Duus, Louise Ayoe Sparvath Brautsch, Andreas Jørgensen, Camilla Thørring Bonnesen, Rikke Fredenslund Krølner

**Affiliations:** National Institute of Public Health, University of Southern Denmark, 1455 Copenhagen, Denmark; titt@sdu.dk (T.T.-T.); ksdu@sdu.dk (K.S.D.); loas@sdu.dk (L.A.S.B.); ajor@sdu.dk (A.J.); catb@sdu.dk (C.T.B.); rikr@sdu.dk (R.F.K.)

**Keywords:** sustainability, public health interventions, physical activity, high schools, qualitative research

## Abstract

Ensuring the sustainability of school-based public health intervention activities remains a challenge. The Young and Active (Y&A) intervention used peer-led workshops to promote movement and strengthen students’ sense of community in 16 Danish high schools. Peer mentors inspired first-year students to implement movement activities. To support sustainability, we applied a three-year stepwise implementation strategy using university students as peer mentors in year 1 and senior high school students in the following two years. This study explores the sustainability potential of Y&A, focusing on school coordinators’ reflections on the intervention’s fit to their schools and the student-driven approach, and we assess the three-step implementation strategy. The study is based on telephone interviews with coordinators (n = 7) from schools that participated in all three years and participant observations of four workshops (a total of approximately 250 participating students). Results were generated through an abductive analysis. Seven schools continued the intervention throughout the three years and adapted it to fit their priorities. The student-driven approach was perceived to be valuable, but few student-driven activities were initiated. Teacher support seemed crucial to support students in starting up activities and acting as peer mentors in workshops. The three-step implementation strategy proved valuable due to the peer-approach and the possibility of gradual adaptation. In future similar initiatives, it is important to address how the adequate staff support of students can be facilitated.

## 1. Introduction

Low physical activity (PA) levels among children and adolescents and decreasing PA levels during adolescence remain a world-wide public health challenge [1,2,3,4,5]. In Denmark, 84% of 16–18-year-old high school students do not meet the national PA guidelines [6]. Few school-based interventions have targeted PA in this age group, and these have produced inconsistent results [7,8]. Furthermore, school-based intervention activities are seldom sustained after the termination of the formal research project [9,10,11,12,13].

In general, research into the sustainability of public health interventions is challenged by an unclear conceptualisation of sustainability and thus the ability to assess to what extent sustainability has been achieved in a research project [14,15,16]. The very notion of sustainability seems to rest on different perspectives on the role of evidence-supported interventions in the real-world: whether the focal point of interest is the intervention or the context into which the intervention is implemented [17]. The different perspectives include viewing sustainability as an end goal or as a continuous process [16]. Moore et al. (2017) and Lennox et al. (2018) have recently reviewed studies using different definitions of sustainability and classified the described sustainability dimensions into the following cross-cutting constructs: continuation of programme activities, continued health benefits for individuals/systems, capacity building/maintenance of implementer behavior change, further development/adaptation, recovering cost and time [16,18,19]. 

According to reviews by Shelton et al. (2018) and Lennox et al. (2018), sustainability is increasingly perceived as a dynamic concept, reflecting a process rather than an end goal [14,16]. This perspective implies that adaptations of interventions during implementation are considered necessary and valuable to support the intervention’s fit to the complex and evolving nature of real-world contexts [14,20]. This point is also reflected in the further development/adaptation construct described above. However, more research is needed on the character of these modification processes, including decisions on discontinuation [14,17,21]. The types of adaptations needed to improve the implementation and sustainability of an intervention in a specific context have been insufficiently investigated. 

In line with the perspective of sustainability as a process that needs ongoing attention, experts within intervention research and frameworks such as the RE-AIM framework advocate strongly for early planning of sustainability. While many studies identify barriers and facilitators for the sustainment of interventions, few studies have evaluated the working mechanisms of planned sustainability strategies [12,14,15,19,22], such as training and capacity building, or a staged approach to implementation [12]. 

Student participation is considered essential for the implementation and sustainability of health-promoting initiatives in schools [12,23,24,25]. Moreover, student participation seems to enhance student outcomes (e.g., motivation and PA), school organisational outcomes and social relationships in school [26]. However, the target group’s involvement and support for the intervention in relation to sustainability has received limited attention within existing sustainability research [12,16], and student participation as a sustainability strategy is an area of future research [12,23]. Thus, we do not know if students are able to play—and interested in playing—a role in sustaining school-based health-promoting initiatives.

Within a Danish high school setting, studies on how to support the sustainability of health promotion activities are lacking. The Young and Active intervention (Y&A) was developed to promote movement throughout the school day, increase well-being and strengthen peer relations among first-year high school students in Denmark. Early considerations of approaches to facilitate implementation and sustainability were part of the intervention development process. To facilitate the sustainability of the intervention, a three-year stepwise implementation strategy was applied [27]. The strategy implied a gradual hand over of the intervention from the research team (year 1) to the students and staff (year 3), supporting a student-driven intervention approach and allowing each school to adapt the intervention to their local context and student population. The aim of this study is to explore the potential of these sustainability strategies. First, we examine why some schools did not wish to continue the intervention in year 2 and 3. Second, among those who did participate, we explore school coordinators’ reflections on the intervention’s fit to their specific high school, the adaptations needed and whether they considered the continuation of intervention activities after the research project ended. Third, we explore the potential of student participation as a sustainability strategy. Thus, the study contributes to an evaluation of the three-step implementation strategy. Relating to the sustainability constructs [16,18], this study explores the imprints left by the intervention (continuation of programme activities) after three years (time) considering the student-driven approach (capacity building/implementer behavior change) as well as adaptation.

## 2. Materials and Methods

### 2.1. The Young and Active Intervention

Y&A was one of four intervention components that aimed at promoting well-being among high school students in Denmark in the Healthy High School intervention (HHS). HHS was tested in a cluster-randomised trial including 16 intervention and 15 control schools [28]. 

In the beginning of the school year, a peer-led innovation workshop was facilitated at each intervention school to inspire first-year students to invent and plan new movement activities which could take place at school before, during and after school hours, preferably on a regular basis. The first-year students were responsible for implementing the activities at school during the school year [27,29]. Thus, Y&A comprised both the workshop and the student-driven activities following the workshop. In year 1, the high schools implemented activities such as a hockey tournament, a canoe trip and yoga classes and purchased new facilities such as a climbing wall, tables for table tennis and smaller sports equipment as goals, balls and bats. The numbers of implemented activities and facilities varied considerably among the schools. Most of the activities were characterised by being short-lived events, while the facilities could be used on a regular basis. In year 2, we did not register the activities systematically. 

#### Sustainability Strategies of the Young and Active Intervention

A central element of Y&A that was presumed to support implementation and sustainability was student participation, both in relation to conducting the workshops (peer-to-peer approach) and as primary drivers of developing and implementing the movement activities invented at the workshops (student-driven activities) [12,25]. In the project communication to schools, the involvement of students was emphasised to ensure the intervention activities appealed to students and to decrease school staff’s workload in relation to the intervention, as a lack of time is a common barrier for the implementation of school-based interventions [10,12]. Emphasis was also put on communicating a broad concept of movement that appealed to all students [30], including those who usually do not take part in school sports. Thus, the intervention was assumed to initiate an inclusive and engaging process of activity development and to build up a student-driven culture of movement. 

To promote sustainability, the research group planned to gradually hand over the delivery of the 3-hour workshop to the schools in a 3 year project period. In year 1, we recruited university students in Sports Science and Health to act as peer mentors. The university students used movement-based innovation methods [29]. In year 2, the workshop was split into two parts of each 90 min. The university students facilitated the first part in collaboration with senior high school students (new peer mentors) and teachers, and the high school students and teachers were responsible for facilitating the second part. In year 3, the high schools were responsible for delivering both parts of the workshop without the involvement of the university students. By training the students and teachers to conduct the workshop themselves, we aimed to support capacity building at the school [14,31]. 

We targeted first-year students as we intended to introduce a norm of movement early in the students’ three-year high school period with the aim of creating a school culture of movement. We also strived for institutionalisation [31] through encouraging high schools to continue conducting workshops for the students of coming year groups, ensuring that activities continued to match the current student population’s preferences. 

In year 1, the students at each school could apply for a start-up grant of 40,000 DKK (equaling approximately 4800 GBP) to support the purchase of new equipment and the establishment of facilities. In the second and third year, the schools received an email with tips, implementation manuals and tools for planning and facilitating the workshop and supporting students’ initiation of new activities, including PowerPoint presentations, graphic materials and an inspiration catalogue. Together with the new equipment and facilities purchased in year 1, this workshop material was assumed to represent sustainable intervention elements. Depending on future students’ interest, the new school activities were in principle elements that could be continued forever.

### 2.2. Data Collection

We employed qualitative methods to explore the potential of the sustainability strategies at intervention schools, including school coordinators’ perspectives of valuable and challenging elements of the intervention [17,32,33]. The coordinators had been responsible for planning the continuation of Y&A in year 3, and we therefore assumed they had an overview of the actual activities implemented and knowledge about the decisions related to the planning process. Furthermore, the coordinators had experienced how the intervention had unfolded throughout the 3-year period and therefore could contribute with a perspective on the 3-year implementation process as a whole. The students, on the other hand, had only experienced the year in which they participated in the intervention as first-year students or the years in which they had been workshop facilitators [28]. Therefore, to explore the sustainability potential at this stage, we chose to build the study primarily on coordinators’ perspectives.

Before the start of the third project year (school year 2018/2019), we contacted all intervention schools to get an overview of their commitment to Y&A that year. At seven high schools, staff intended to conduct the workshop, or another activity inspired by Y&A, among first-year students. In the period from November 2018 to January 2019, we interviewed school coordinators from these seven schools (Table 1). The coordinators comprised two head teachers, two mid-level managers and three teachers (two in physical education (PE) and one in Danish/religion). Except for one of the PE teachers, all coordinators had been involved in the project from the beginning (2016/2017). 

Throughout the project period, the schools participated in various kinds of data collection, and we therefore aimed to minimise further “disturbances” by the end of the project. As school staff work under tight schedules and a high degree of contact had been established earlier, we chose telephone interviews as a flexible, practicable and viable option for conducting semi-structured interviews [34].

The interviews lasted on average 26 min and were based on an interview guide covering the themes of the form and content of the workshop, the facilitation of implementation processes related to the student-driven activities, appreciation, fit to existing priorities and considerations regarding continuation. The interviews were recorded and transcribed verbatim by research assistants who were not part of the project group. The transcriptions were based on a detailed transcription guide. 

To capture the local adaptations of the workshop and explore the implementation of the student-driven approach, we supplemented the coordinators’ perspectives with participant observations of four workshops covering three schools located in different geographic areas (Table 1) [33]. The participant observations were conducted by three of the authors, in some cases simultaneously. At one school, we observed both parts of the workshop. We produced descriptive and reflective field notes [35].

To investigate the reasons for not continuing Y&A in year 3, we used previously collected data including emails from coordinators stating their reasons for withdrawal as well as coordinator interviews conducted during year 1 as part of the overall process evaluation of the HHS [36].

### 2.3. Analysis

The first author, S.K.W., conducted the analysis as an abductive process moving between the empirical material, established assumptions of the research project (programme theory [29]) and existing literature [32,37]. In short, this analytical procedure implied the following concurrent methodological steps: several thorough readings of the empirical material, continuous consideration of existing knowledge and theory (e.g., the research team’s pre-established assumptions about mechanisms and the dynamic concept of sustainability), extensive writing and re-writing and collaborative analytical reflections with co-authors [38]. S.K.W. scrutinised the material several times, starting exploratively with the question “What is this a case of?” in relation to the sustainability potential of the intervention [37]. This process resulted in the identification of two overall themes: adaptation and the student-driven approach. In the following steps, these themes were elaborated and coded based on both existing themes of the interview and observation guides—e.g., workshop facilitation—and new themes identified during the analytical process—e.g., local meaning. Implementation literature on sustainability also formed the analytical lens; for example, in relation to the complex and procedural character of the implementation process and the significance of teachers as an important stakeholder group [11,12,14]. To refine the analytical insights and ensure communicative validity [39], the preliminary analyses and results were continuously discussed with co-authors, some of whom had participated in the data collection and some of whom were unfamiliar with the intervention project [38].

## 3. Results

The results are summarised in Table 2.

### 3.1. Continuation of Young and Active in Year 3

In year 1, the workshop was conducted at 14 schools, as two schools decided not to implement the intervention upon randomisation. In year 2, workshops were conducted at 10 schools; 6 of these conducted both parts of the workshop. In year 3, half of the schools (N = 7) conducted the workshop, or an event inspired by Y&A (Table 3). Three schools conducted both parts of the workshop. At two schools, the Y&A concept was modified considerably. 

Primary reasons for not continuing the workshop after year 1 were time and resource constraints related to the preparation and implementation of a new educational reform in the school years 2016/2017 and 2017/2018, and spending cuts of 2% annually since 2016. Furthermore, one school did not find the initiative useful, while two other schools did not provide a reason. At one school, the management assigned the task of organising the workshop in year 3 to the student council, who did not take action. 

### 3.2. A Process of Fitting

The qualitative study showed that the schools adapted the aim and the facilitation of the workshop. Below, we show how the potential sustainability of Y&A concerns a multi-faceted process of fitting the intervention to the schools’ educational and organisational structures and priorities through coordinators’ reflections on experienced investments, new discoveries, and perspectives. 

#### 3.2.1. Modified Aim and Focus of Workshop

Student well-being was a high priority at all schools, which was therefore compatible with the aim of the workshop. At the majority of schools, the management seemed to support the implementation and continuation of Y&A. 

At some schools, only few activities had been initiated after the workshop in year 1; hence, some coordinators did not find the workshop useful after the first year. The innovation process had been too open, and many of the students’ ideas had been unserious and concerned parties and alcohol consumption. Through the three-year implementation process, some coordinators experienced a sharpened focus and purpose of the workshop due to the research group’s minor refinement of the form and content of the workshop for year 2 and the schools’ own selection of certain domains they wished to prioritise, such as the development of activities for the breaks. 

At most schools, creating a strong sense of community among students was an explicit purpose of the workshop in year 3. For example, one coordinator stated the following:

*As a private high school and as a small high school, we very much depend on a great feeling of well-being among our students. Also, across year groups. We don’t want the third-year students to hate the second-year students, or something like that, or the other way around, because we are so close to each other. So, we must try to mix them a bit. And that’s why it [Y&A workshop] has been a really good opportunity for us to get the third-year students and the second-year students a little interested in the first-year students*.

(School C)

At some schools, promotion of movement was not equally prioritised, probably because many movement activities existed already, partly as a result of the workshops conducted in year 1 and 2.

In year 3, the workshop seemed to meet a new need of the schools created by the new educational reform, which restructured the study programme by postponing the establishment of the final specialised study programme classes to three months after school entry (November). Many of the coordinators described this change as challenging for the students, because they spend a large amount of energy during their first months at school getting to know their classmates in the temporary classes to which they are allocated to at school entry (August) and then have to start all over again in November. Therefore, the school staff prioritised the creation of a safe start and sense of community in the new study programme classes. Three of the schools found the workshop useful for this purpose.

The observations illustrated three ways in which the schools integrated the workshop into their timetables. At one school, the workshop formed part of PE; at another school, the workshop formed part of a one-day introduction course just after the formation of new study programme classes; and in the last case, the workshop was scheduled in a time slot normally dedicated to voluntary sports or music classes (occurring one morning every week). These different ways of integrating the workshop in the school’s schedule might have implications for the signal sent to the students, i.e., the degree to which Y&A was perceived a mandatory part of established teaching (PE) or as more of an addition. 

The observations also illustrated how the facilitation of the workshop to different degrees was based on the manual and materials. At one high school, the manual was used slavishly, and the facilitators provided the students with all the materials. At the other two schools, the manual was not equally followed, but central elements and principles, such as the body-based innovation exercises, were evident.

#### 3.2.2. Attentional Investment and New Perspectives 

As illustrated in Table 3, all schools intended to continue the specific Y&A initiative or its underlying principles after the conclusion of the research project. The coordinators expressed that it had been a three-year process of investing attention and experiencing progress, which now acted as a motivator for continuing the initiative. For example,

*We already have a strong focus on well-being, and it’s a good way to get the students self-enrolled in the well-being idea by having them arrange it the year after, by making the senior students the ones to actually make sure that the first-year students thrive … (…) So we certainly think this has become a focal point over the last many years … Now with this, we have started to make things move, and, well, we’ll continue*.

(School C)

Lack of time and work pressure on teachers and managers (partly due to the reform and spending cuts) was recurrently mentioned as a barrier for implementation. Therefore, a plausible interpretation of the illustrated perspective of investment is that the work of three years should not be wasted. This attention to not wasting time was also apparent in the coordinators’ initial scepticism about the workshop:

*And then it has also been important to make sure that we can use this for something, that it’s not just 90 min that have been wasted and so on. That it has some usefulness and value in relation to what we can use it for as a school*.

(School E)

At two of the observed workshops, we noticed that the coordinators and peer facilitators referred to the activities and experiences of the previous two years of Y&A, which illustrates their awareness of and emphasis on the procedural and developmental character of Y&A. At the third school, the responsibility of the workshop had been delegated to a new teacher, who could not refer to the experiences of previous years. The replacement of central stakeholders, then, has implications for perceiving and working with Y&A in a developmental manner.

Some of the coordinators explained in the interviews how Y&A had prompted new insights; for example, that new high school students can bring fresh energy and a wish to contribute to the school community if they are given the opportunity:

*It’s probably this culture of volunteering and doing things together that has become really strong, among other things, through the project here*.

(School F)

The intervention had provided new inspiration for working with students’ well-being. For example, two coordinators described that they would like to initiate an innovative process in which students should reflect on their own perspective of the concept of well-being, including aspects relating to use of social media. Some coordinators also mentioned that the innovative methods had given new didactical inspiration.

Y&A and the larger HHS project also increased the attention of some coordinators’ attention to a broader mission of high schools:

*I do think that it has created some awareness of the importance of having a school life that’s more than attending classes, and that it’s not good for the students to just be sedentary for a whole day, but that they must also be actively challenged, and that we focus on the fact that there’s an overall perspective, that it’s more holistic.... That we have realised that we are more than a centre of knowledge. And that knowledge is better embedded if the other elements are included*.

(School E)

When asked to describe the gains of the initiative, the coordinator from school C replied as follows:

*I think we need a focus on play and joy in the educationally heavy everyday life, which characterises STX [an upper secondary education programme lasting 3 years] and HF [an upper secondary education programme lasting 2 years].*

### 3.3. Exploring the Sustainability Potential of Student Participation

The analysis shows that it was challenging for the students to perform the various tasks implied in the initiative by themselves. The involvement of teachers or a school manager is important in both conducting the workshop and the initiation and sustainment of activities derived from the workshop.

#### 3.3.1. Student-Driven Intervention Activities

In year 3, the workshop for first-year students was facilitated by senior students at most schools, as suggested by the research group, with support from staff—often the coordinators (Table 3). Only one school did not use peers as workshop facilitators.

Several coordinators regarded the peer-to-peer approach as valuable for first-year students, senior students and teachers. One coordinator stated the following:

*Our intro tutors are really happy that …, well, they also love being in the spotlight themselves, so it’s kind of a win-win that the new ones they want … they also love to meet someone at eye level … So in that way, I think we have found a model that runs relatively resource-free, it doesn’t require that many resources in time and teachers, etc.*

(School F)

During the participant observations, we witnessed that the peer-approach facilitated a safe and eye-level relation between the first-year and senior students. For example, in general, the senior students seemed comfortable with their role as facilitators, and they were able to guide the first-year students through the exercises. They also created a relaxed atmosphere using background music and by joking with the first-year students, but at the same time they were able to point a direction and communicate the aim of the exercises in a serious manner. However, we also saw situations in which the atmosphere became too relaxed and where the senior students momentarily let go of the facilitating role; for example, in one situation, pictures from a recent school party were suddenly released, stealing all the students’ attention. 

These observations were in line with the coordinators’ perspectives. They stated that the first-year students looked up to the senior students, and the workshop created an opportunity for meeting at eye-level that facilitated the creation of relations across year groups. Many coordinators found that the senior students facilitated the workshop with great energy. Moreover, they believed it was inspiring for the first-year students that the senior students had volunteered for the job. 

During the participant observations at two high schools, the senior students facilitating the workshops told us that many other senior students had shown interest in becoming a workshop facilitator. According to some coordinators, the workshop facilitation represented a chance for the senior students to be in the spotlight and to get an instructive teaching experience:

*… I also think they have gained a lot from trying to be in a teaching situation… To be on the other side … instead of having to receive messages, then having to give them to 90 first-year students who don’t always listen, and so on. So, I think they have gained a lot from this*.

(School B)

All coordinators agreed that it was challenging for the first-year students to initiate the activities developed at the workshops by themselves. At most schools, only few entirely student-driven activities had been initiated during the first project year. They recognised that not all students are interested in and willing to participate in developing activities. One coordinator expressed how the workshops in the first year had ended in dead ends:

*Well, the students, once they had their ideas, they were not particularly keen on moving on with them. They didn’t really think their ideas would stick. So, I think we reached a dead end many times. We had one or two successes with something that got started and could be realised. Well, about taking initiative …, I think there is something in the fact that you are responsible for going further with something. Something fails there*.

(School G)

The coordinators mentioned that students have many other things to think about, especially upon high school entry, where new friendships and routines are being established: “Eventually, everyday life sets in”, and the students forget about the workshop and their ideas for activities. 

Based on the research group’s experiences of the implementation challenges of previous years, it was decided to make the second part of the workshop optional for the students in year 3. Some high schools adhered to this to ensure that only students with interest in developing activities participated. 

Some coordinators described the presence of program champions among students as important for the development and implementation of activities:

*Some of the students are really passionate, sometimes they can drive an activity for a long time, their entire high school time. Sometimes the activity stops because certain students graduate, and that’s the course of nature for high schools*.

(School F)

*We had some students who for about a year and a half had an Ultimate division (which was initiated as a result of the Y&A workshop and start-up grant in year one), where they played both summer and winter at the school. A good, solid group of 10–15 young people, and they had fun and had a great time, but they also ran it themselves pretty much. And not everyone is lucky to be part of a class with people who are drivers or take on this responsibility naturally—people who are willing to do it, are capable of it and bother*.

(School G)

#### 3.3.2. Staff Support in Workshop Conduction

Several coordinators recommended that the overall responsibility of the workshop was delegated to themselves. For example, one coordinator expressed:

*When you start something like this, I feel it is necessary to have a backup plan, that is, if students [senior student facilitators] do not show up … you have to get involved, there has to be someone who’s mature enough to take responsibility, to make sure that things are going to work*.

(School C)

However, several of the coordinators experienced that senior students *were* able to facilitate the workshop after the practicalities of the workshop had been organised (room booking, procurement of equipment, etc.) and the students had received instructions:

*But when the framework is established and people have turned up, it runs perfectly through student glasses and student eyes and student facilitation*.

(School C)

*They cannot do it themselves 100%, nor should they (the student facilitators). But then again, they could actually, with a little help*.

(School E)

Apart from arranging the practicalities of the workshop, the coordinators described in various ways how they had supported the students and prepared them for facilitating the workshop. One coordinator described how they supported the senior students by instructing them in how to appear with natural authority as facilitators:

*And of course, they have to work on—and this isn’t natural for everyone, and for some it’s very natural—this authority that a teacher can have. But we talked about that … Well, that’s also why it was a good idea that I was there, and my colleague, too, we talked about how to create authority*.

(School E)

Another coordinator believed that he had an important role in showing a direction for the student facilitators; for example, stating that the aim of the workshop should be to develop active breaks for lessons. In the previous years, he had experienced the brainstorm activity to be too unstructured, resulting in few good ideas for activities. He explained his perspective of appropriate work-sharing:

*It’s important to keep the product in mind, and it requires taking a little control. It can get very open without control. The students have a lot of ideas, and they are the ones you have to get hold of because they are the ones who have to live with the activities, but one person should take charge, take control and provide structure, for example focus on breaks or active breaks for lessons. Students lack routine. But they are good at being in the spotlight and at the creative process*.

(School C)

The senior students’ lack of teaching routine was also seen during the observations; for example, in situations in which they became lost in the agenda of the workshop and when the situation became too relaxed, as mentioned above.

One coordinator indicated the didactical value of the workshop, which provides an opportunity for teachers to learn how students work in innovation processes. He believed that some of the school’s teachers ought to attend a workshop to carefully observe how the first-year students worked during the innovation processes and try to bring some of these observations into teaching. He believed it was important to work with the initiative in a procedural manner, instead of approaching the workshop as a one-time event and developing ”an activity for the sake of the activity” (School G). 

The coordinators had different perspectives on the necessity of involving other teachers than themselves in the preparation and conduction of the workshop. Using students as intervention providers was perceived as suitable by some coordinators because it reduces the teacher burden, and many teachers do not see these kinds of extra-curricular activities as part of their job. Other coordinators stressed the importance of creating a sense of ownership among the participating teachers to allow the initiative to take root in the organisation.

#### 3.3.3. Staff Support in Activity Development

The coordinators generally recognised the importance of supporting the students in transforming the ideas from the workshop into real activities and “keeping the students’ noses to the grindstone”. However, in most interviews, they only offered reflections on hypothetical scenarios instead of descriptions of actual implementation support. 

At two high schools, the coordinator summarised the ideas generated at the workshop in a document and circulated it on the high school’s digital platform. The purpose was to inspire interested students (or teachers) to initiate an activity and to inform all teachers of the ideas. However, this effort did not result in the initiation of new activities. At two schools, there was no follow-up process due to lack of student engagement in the second part of the workshop or because the school did not organise the second part.

The follow-up process at School F stood out from the other schools as 30 students participated in the second part of the workshop. Here, they signed up to be part of arranging the specific activities, and the further development and initiation of the activities was done in a close collaboration between the students and the coordinator. The coordinator was aware of the students’ need for reminders and support and regularly asked the students whether their ideas were progressing and supported them if necessary. 

While some coordinators in hindsight commented that they probably should have supported the students more, they also believed this would counteract the philosophy of the Y&A initiative:

*But maybe you should have spent 10 min in a teaching session on “Make a Facebook event and then get this up and running”. But I also think it should come from themselves, right? (…) where they show that they have … “This could be super cool, to get this up and running” … This is the point of it all, I think*.

(School B)

*I would like the teachers to take a bit more control, but it will be … then you have to pay them and then you have to… you know, then it has to be included in their work tasks, and that’s kind of against the thinking behind these things*.

(School F)

Some coordinators described that their students had adopted a practice of reminding the teachers of active breaks, which indicates a kind of mutual support. 

The research group encouraged the school staff to support the senior students’ workshop facilitation as well as the students’ initiation of new activities, but without further specification of how this support could be put into practice. Unclear communication about the appointment and sharing of responsibility for conducting workshops and initiating activities, as well as questions about teacher capacities and mandates, emerged as crucial themes for the realisation of the specific activities.

## 4. Discussion

This study explored the sustainability potential of Y&A and found that (1) half of the 14 intervention schools did not proceed with the workshop in year 2 and 3 mainly due to spending cuts and a new educational reform, (2) schools that did continue Y&A in the following years adapted the initiative to fit their specific priorities—for example, by focusing only on strengthening the sense of community—and (3) the coordinators found the student-driven and peer approach valuable in several ways. The fact that many intervention schools conducted the peer-led workshop after the researchers gave up control shows that the three-step implementation strategy has some promise. It seemed crucial to involve school staff in supporting the students in facilitating workshops and implementing their ideas for the activities generated at the workshop. The coordinators had different views on the optimal level and type of staff involvement. The findings indicate that the intervention left an imprint on the participating schools in various ways besides the workshop, activities and facilities; for example, by increasing the staff’s attention to the educational value of working with student well-being and by providing new didactical inspiration, such as peer-approaches and innovation processes. Hereby, the study has identified and covered a breadth of sustainability constructs, namely the continuation of programme activities, capacity building/implementer behavior change and adaptations [16,18].

Outer contextual factors have been found to be of particular importance in intervention sustainment, and this was also the case in Y&A [22]. However, the changes in the outer policy context (the educational reform and spending cuts) applied to all intervention schools [14]; this being the case, why did some schools choose to continue for all three years? This may be explained by Rogers’ [40] five attributes by which an innovation as perceived by the involved individuals influences adoption rates: *compatibility* with existing values, the innovation’s *relative advantage* compared to existing practice, the *trialability* of an innovation on a limited basis, perceived *complexity* of the innovation and the *observability* of the innovation’s results. Although schools’ implementation of the intervention was hindered by tight work schedules and the priority of standard curriculum activities, as seen in other studies [11,12,22], the continuance of activities, procedures etc. indicates that schools experienced a degree of *compatibility* between the aim of Y&A and their own priority for student well-being [40]. Furthermore, school staff seemed to perceive a *relative advantage* of adding Y&A to existing practices in order to meet the evolving need of creating a sense of community in new study programme classes [40]. Attention to this need was also raised by an evaluation of the reform [41]. The three-year process allowed the schools to try out the intervention and accumulate experience with implementing it. Initially, some schools found the intervention to be of less use, causing one school to withdraw. However, some schools continued the intervention despite initial scepticism and adjusted it; thus, it gained value locally. This illustrates the significance of *trialability* for intervention adoption [40]. According to Rogers, the adoption of an innovation is more difficult when the adopters perceive the innovation as *complex* and hard to use [40]. The local adaptions, including a narrowed aim of the workshop at some schools, made it less complex to implement in year 2 and 3. Finally, some of the outcomes of the workshop were readily *observable* for the school staff, such as bonding between first-year and senior students, and senior students’ instructive experiences of facilitating workshops, which probably also motivated continued implementation [40].

In line with the conceptualisation of sustainability as a dynamic process [20], we found that the adaptation of Y&A was inevitable and necessary for the intervention to fit the specific school organisation (structure and priorities) and the changes in the outer policy context. This fitting process takes time, and we saw that the staff’s learning processes and the experience of attentional investment in new things are an active process of value creation. Intervention schools committed themselves to the three-year project by entering the study and could not afford to spend time and energy on something that did not make sense to their overall job of educating young people. Overall, we may consider these processes as “mutual adaptation”, as described by Stirman et al. (2012) [17]. The intervention’s form and content were adjusted to the high schools’ priorities and structures, but the intervention also promoted new insights and practices among the staff, indicating a mutual adaptation as part of a dynamic sustainability process.

Based on the participant observations and the interviews with the coordinators, peer-led workshop facilitation seemed to be valuable for both first-year students as well as senior students recruited as facilitators, but the senior students needed support from teachers. The coordinators found that not all students were interested in or able to participate in starting up new activities by themselves, which is in accordance with the students’ perspectives of Y&A in year 1 explored in a previous study [29]. Mainly, champions among first-year students volunteered to initiate and run activities. Staff support therefore seemed crucial in both types of student-driven intervention activities in order to support the sustainability of the initiative. This is in line with previous studies into the sustainability of school-based interventions [11,12,22,23,42]. Hence, a student-driven approach does not only concern the students but also the teachers and their expertise and eye for educational opportunities. We see that the coordinators in our study had different perspectives on the nature and purpose of teacher–student collaboration. For example, some perceived students’ involvement as helpful to the teachers, sparing them time and workload in an already crowded teacher schedule [23], while others highlighted teacher support to the students’ implementation of activities as important for the students’ general education. 

In line with other studies, we found that head teachers’ and teachers’ genuine belief in and work for the integrated purpose of promoting well-being and education is crucial for implementation [12,25,43]. At some of the schools, this perspective seemed to be underway; for example, in the notion of teaching senior student facilitators how to teach as part of workshop preparation. The overall aim was to support an initiative promoting student well-being, but the teachers also integrated educational elements. However, this perspective was only recurrent at one school (F) throughout the entire process of activity development, continuously resulting in new activities. School F was an exceptional case, as the coordinator’s position as a mid-level manager explicitly included the task of developing the social life at the school. Thus, the implementation of Y&A was perfectly compatible with the coordinator’s job. Based on this and his continuous enthusiasm, he can be viewed as a staff champion [23]. We have even been informed that school F also conducted workshops in year 4 after the conclusion of the research project. Hence, in Y&A, capacity building in applying the peer-to-peer approach locally as well as the gradual handing over of the intervention to students (and school staff) also includes the personal development of students supported by staff, which resonates with the task of general education [25,44].

In line with the existing knowledge within school-based interventions, commitment and support from the management was an overarching facilitator of implementation and sustainability in our study [11,12,22,43,45]. Specifically for school-based interventions, strong leadership seems to be important for articulating a clear mandate to the teachers to work with health promotion within their task of education. Future interventions in this field should encourage a collaborative awareness and belief among stakeholders in the overlapping and mutually supporting aims of health promotion and education [12,43].

Although clearer conceptualisations of intervention sustainability have been developed [18], some challenges remain in assessing sustainability from a dynamic perspective. Referring to the various sustainability constructs described in the introduction [16,18], a central question is the following: what do we—as intervention researchers—expect and assume regarding sustainability? In Y&A, 7 out of 14 high schools continued to conduct workshops or events derived from Y&A throughout the three-year period, which we find to be a quite positive finding, considering the general challenges related to time and resource constraints at schools. This study does not investigate the maintenance of intervention outcomes at the student level. One may ask how a three-hour (mandatory but extra-curricular) workshop can be expected to create any change in movement, sense of community and wellbeing. As mentioned previously, we did not expect the workshop to change these student outcomes in itself. Rather, it was the resulting activities derived at the workshops which we expected to be offered on a regular basis throughout the school year to influence the student outcomes. Thus, Y&A was designed and communicated as a structural initiative facilitating new students being given time, space, inspiration, and support to be physically active at school together with their peers, involving students in developing the social life at school and ensuring access to activities students enjoy [46]. As illustrated in this study, some schools were aware of this procedural nature of the intervention as well as of the investment needed to implement new initiatives, while other schools probably saw it as a one-time event. We consider adaptation as a necessary part of the complex process of rooting new health promoting practices and principles in a school context [12,14], but it remains to be explored how much adaptation is “allowed” to produce the desired outcomes of the intervention. In this case, the overall aim was to promote well-being through movement and an increased sense of community, and in several cases the aim of promoting movement was eclipsed by the high schools’ priority of strengthening a sense of community. 

Through an exploration of the research project’s substantiated assumptions of sustainability, this study has contributed to a nuanced and contextualised understanding of valuable intervention sustainability strategies within a Danish high school setting. Specifically, the study has offered an evaluation of how the intervention was continued at the high schools, how it was adapted and how the process of handing over the intervention to students and school staff through a novel three-step implementation strategy (capacity building) unfolded at different interventions schools. Through the study, additional questions and curiosities have arisen; for example, on how to nurture student–teacher collaboration in extra-curricular activities, calling for research to follow and further develop initiatives such as Y&A. 

### Strengths and Limitations

According to some scholars, exploring and adjusting implementation strategies takes priority over studying effectiveness when the intervention is complex and implemented in a complex real-world setting [47]. An important strength of Y&A is the three-year stepwise implementation strategy, which allowed us to study the transition from researcher-driven intervention delivery in the first project year to the adoption and possible sustainability of the initiative in the third project year [14,29,48]. The qualitative approach enabled us to explore a breadth of sustainability dimensions, including unexpected imprints such as the value of the intervention in relation to the new educational reform, and to offer a nuanced account of the complex sustainability process [16].

The present study primarily represents the perspectives of the coordinators, which can be viewed as a study limitation. It would have been fruitful to include the senior students’ perspectives of the three-year process to a larger extent to provide nuance to the findings. However, as students already had contributed extensively to both qualitative and quantitative data collection activities related to other aims of the HHS study throughout the three-year period [29,36,49], we were reluctant to disturb them once again. We supplemented the coordinators’ perspectives by including participant observations of workshops as a third-person view on the senior students’ engagement in the workshop facilitation. The workshops or events inspired by Y&A at the seven intervention schools were, however, scheduled in a way that only allowed us to observe four workshops representing three high schools. The composition of the group of authors, including some who were familiar with the project and one experienced researcher who was unfamiliar with the project, was a strength in the collaborative analytical process, as the various perspectives enriched the analysis and supported the communicative validity [39].

Telephone interviews present both advantages and limitations [50]. In this study, we noticed that several interviews proceeded around a particular “script”, where coordinators framed smaller critical remarks positively, leaving the overall impression of high intervention satisfaction. The way people communicate on the telephone may have influenced the degree to which the participants elaborated on critical points; for example, it is difficult to interpret breaks in the telephone conversation involving a risk of disrupting the participants’ reflections [51]. However, the interviews *did* include critical points. In addition, the supplementary perspective from the observations and previous insights from the intervention study provided a valuable “canvas” for interpretation.

## 5. Conclusions

In line with the recommendations on how to sustain public health, the Y&A intervention was from the beginning developed with strong considerations on how to support the sustainment of the intervention. This qualitative study responds to a call for evaluations of planned sustainability strategies. The study explored these sustainability strategies with a particular focus on student involvement and adaptation and thereby responds to scholars’ call for studies evaluating the working mechanisms of planned sustainability strategies [12,14,15,19,22]. Our study builds on a dynamic perspective on sustainability [20] and offers a nuanced account of the imprints left by the intervention as well as adaptations supporting the sustainability of Y&A. Specifically, the study explored reasons for schools’ withdrawal, school coordinators’ reflections on the adapted interventions’ fit to their specific high schools and the planned sustainability strategy of letting the students lead the initiative, which was supported through a three-step gradual hand-over of the intervention. The three-step implementation strategy was a new example of how to plan for intervention sustainability, and it allowed us to explore the complexity of delivering and handing over an intervention intended to be received and sustained by the target group of high school students. The peer-approach and the three-step implementation strategy showed some promise and might be a valuable strategy for future school-based interventions aiming to support sustainability. However, the approach depended extensively on different types of teacher support. For future interventions in high schools building on similar participatory principles and aiming for sustainability, we suggest researchers, school management, teachers and students work together to consider how an adequate staff support of students can be facilitated. In this respect, it seems important that researchers and staff collaboratively address and articulate the educational opportunities in health promotion activities.

## Figures and Tables

**Table 1:** Overview of participants in the data collection.

**Telephone Interviews**					
High School	Coordinator’s Position		Gender		Involvement in the Project
A	Head teacher		Female		All three years
B	PE teacher		Male		Only the third year
C	Mid-level manager		Male		All three years
D	PE teacher		Male		All three years
E	Danish/religion teacher		Female		All three years
F	Mid-level manager		Male		All three years
G	Head teacher		Male		All three years
**Participant Observations**					
High school		Approximate number of students participating in workshop		School size and geographical area	
B (workshop part one)		60–70		Small school located in the Capital region	
B (workshop part two)		60–70		Small school located in the Capital region	
C		33		Small school located in the Northern part of Zealand	
E		80–100		Large school located in Jutland	

**Table 2:** Overview of themes and subthemes of analysis.

Main Theme	Sub-Themes	Key Points
Continuation of Y&A in year 3		Half of the high schools continued the intervention throughout the three-year project period. Implementation of a new educational reform and spending cuts were primary reasons for not continuing Y&A after year 1.
A process of fitting	Modified aim and focus of workshop	Coordinators experienced a sharpened workshop focus throughout the three-year period. In year 3, the main purpose was narrowed to support students’ sense of community. Due to the new educational reform, this was perceived as important and the intervention was found to fit this purpose. The way the intervention was integrated in schools’ timetables varied. The degree to which the schools adhered to the intervention manual varied.
	Attentional investment and new perspectives	Investing attention and experiencing progress motivated the coordinators to continue the intervention. Among the coordinators, Y&A prompted new insights, inspiration and a perspective of a broader mission of schools towards promoting students’ health and well-being.
Exploring the sustainability potential of student participation	Student-driven intervention activities	All but one school used senior students as workshop facilitators. Peer-facilitated workshops created a safe and eye-level relation between first-year and senior students. At some schools, program champions among students developed and implemented activities, while only few or none activities were implemented at other schools.
	Staff support in workshop conduction	Senior students were able to conduct workshops with some support from the coordinators; e.g., regarding practicalities or instructions in being an authority. Different opinions existed among the coordinators regarding the involvement of teachers in workshop preparation and conduction.
	Staff support in activity development	In most cases, the students were not supported by teachers or coordinators in implementing activities, although the coordinators recognised the students’ need for support. At the same time, the coordinators found teacher-support to be against the philosophy of the Y&A project (the student-driven approach). Following-up on the workshop and supporting the students’ implementation of activities was successful at one school, where the coordinator offered close and regular support to the students.

**Table 3:** Overview of the implementation of Young and Active (Y&A) in year 3 at intervention schools.

High School	Form and Timing	Purpose	Peer-to-Peer and Teacher Involvement	Plans for Continuation after Year 3
A	A Y&A-inspired event in the beginning of the school year.	Sense of community: To recruit new students for student associations such as the party planning committee and the school magazine	The event was entirely led by first and second-year students, but teachers participated in a short preparatory meeting.	The event will be repeated next year.
B	Two workshops similar to the original workshop conducted during PE lessons in November.	Movement and sense of community	Third-year students of PE (elective course at higher level) planned and facilitated the workshops in close collaboration with the coordinator (PE teacher).	The coordinator thought the initiative would continue. The school was closed afterwards.
C	Two workshops similar to the original workshop conducted in the beginning of the school year.	Movement and sense of community, especially focusing on active breaks during class and activities for the breaks.	The coordinator (head teacher) planned the workshop and held a preparatory meeting with senior students. At the workshops, he gave the introduction, and afterwards the senior students facilitated the rest of the workshops.	A clear plan of continuing the workshops.
D	One workshop in November as part of introduction to new study programme classes.	Movement and sense of community, especially focusing on events and active breaks during class.	The workshop was entirely led by the coordinator (PE teacher) and another teacher.	The management is keen on the initiative and plans to continue it next year.
E	One workshop similar to the original workshop conducted in November as part of introduction to new study programme classes.	Primarily sense of community, but also movement.	The coordinator held a preparatory meeting with second and third-year students. These senior students facilitated the workshop supported by the coordinator and two PE teachers.	Y&A will continue in some way.
F	Two workshops similar to the original workshop conducted in the beginning of the school year.	Primarily sense of community, but also movement.	The coordinator held a preparatory meeting with second and third-year students. These senior students facilitated the workshop supported by the coordinator.	Clear plan of continuing (and the research group has been informed that the event was repeated in year 4).
G	One workshop in November as part of introduction to new study programme classes.	Sense of community (and a little bit of movement): To let new students produce videos which illustrate the distinctive feature of each school class.	The workshop was conducted by two teachers and two students from the student council.	Plans for continuing with selected principles of Y&A. The head teacher (coordinator) has afterwards (year 4) left his position.

## Data Availability

The data that support the findings of this study are available from the University of Southern Denmark, but restrictions apply to the availability of these data, which were used under license for the current study and so are not publicly available. Data are however available from the authors upon reasonable request and with the permission of the University of Southern Denmark.
